# Association between multimorbidity and memory-related diseases among middle-aged and older adults: Evidence from the China Health and Retirement Longitudinal Study

**DOI:** 10.3389/fpubh.2023.1115207

**Published:** 2023-03-17

**Authors:** Chen Chen, Yihao Zhao, Binbin Su, Yu Wu, Panliang Zhong, Xiaoying Zheng

**Affiliations:** ^1^Department of Aging and Health, School of Population Medicine and Public Health, Chinese Academy of Medical Sciences & Peking Union Medical College, Beijing, China; ^2^Department of Chronic Diseases, School of Population Medicine and Public Health, Chinese Academy of Medical Sciences & Peking Union Medical College, Beijing, China; ^3^Department of Health Economics, School of Population Medicine and Public Health, Chinese Academy of Medical Sciences & Peking Union Medical College, Beijing, China

**Keywords:** multimorbidity, dementia, memory-related diseases, non-communicable diseases, Chinese middle-aged and older adults

## Abstract

**Objectives:**

This study aimed to examine the cross-sectional and longitudinal association between multimorbidity and memory-related diseases (MDs) among Chinese middle-aged and older adults.

**Methods:**

This study included 8,338 subjects who participated in the China Health and Retirement Longitudinal Study (CHARLS). Logistic regression and Cox proportional hazards regression models were used to explore the association and effect of multimorbidity on MDs.

**Results:**

The overall prevalence of MDs was 2.52%, and the mean multimorbidity number was 1.87. In a cross-sectional analysis, compared with the no multimorbidity group, groups of multimorbidity with four or more non-communicable diseases (NCDs) were more likely to have MDs (OR: 6.49, 95%CI: 4.35–9.68). Within 2.7 years of follow-up, 82 cases of MDs (1.12%) were reported, and participants with multimorbidity were more likely to have new-onset MDs than participants without multimorbidity (HR: 2.93, 95%CI: 1.74–4.96).

**Conclusion:**

Multimorbidity is associated with MDs among Chinese middle-aged and older adults. This relationship gradually strengthens with the severity of multimorbidity, which indicates that early prevention for people with multimorbidity may reduce the risk of MDs.

## Introduction

Declining fertility and increasing longevity are the main drivers of population aging globally (1). According to the projection of the WHO, the number of people aged 65 years and above is expected to be 2 billion by 2050 (2). Due to population aging, the prevalence of dementia has increased dramatically from 1990 to 2016, especially after the age of 65 (3). According to the 2022 World Alzheimer Report, approximately 55 million people lived with dementia worldwide in 2019, and it is expected to reach 139 million by 2050, with the majority coming from low-income and middle-income countries (LMICs) (4). A nationwide cross-sectional study reported that there were 15.07 million Chinese older adults (aged 60 years and over) suffering from dementia (5). The primary causes of dementia are MDs among older adults, including Alzheimer's disease (AD), brain atrophy, and Parkinson's disease. MDs are a multisystemic disease, for which effective treatments are still lacking (6, 7). In addition, dementia is the primary cause of disability and medical care needs among older adults (8, 9). Meanwhile, dementia carries a severe medical burden and economic costs. Specifically, in China, the annual treatment cost of treating patients with AD was 167.74 billion dollars in 2015, and by 2050, the treatment cost is expected to reach 1.8 trillion (10).

Non-communicable diseases (NCDs) are the leading causes of morbidity and mortality worldwide (11). With the increasing aging population, older adults are no longer suffering from only one NCD but two or more NCDs simultaneously, which is conventionally termed multimorbidity (12, 13). A recent study has reported that more than 50% of older adults have multimorbidity in high-income countries (13), and this figure is expected to reach 68% by 2035 (14). Considering the complex needs and high medical costs, multimorbidity poses a significant burden on the healthcare system and could seriously undermine financial protection and universal health coverage (15). Because people are getting older and more exposed to risk factors, the burden of multimorbidity is also rising rapidly in LMICs (16–18). In China, a related study reported that the prevalence of multimorbidity was 61.9% among middle-aged and older adults (age ≥ 45) in 2015 (16). Another study found that health expenditure was associated with an increased number of NCDs and that multimorbidity had a great adverse impact on patients' health outcome and healthcare system burden than individual NCDs (19).

Several NCDs, such as diabetes (20), hypertension (21), and cardiovascular diseases (22), have been identified as at-risk conditions for increased dementia incidence. However, older adults often suffer from multimorbidity, and evidence of multimorbidity effects on dementia is relatively scarce (23). Previously, some cross-sectional studies in high-income countries have found that older adults with dementia often concur with multimorbidity (24). As for the longitudinal association, a recent study reported that older adults with multimorbidity had a high risk of dementia (25). Another study also found that multimorbidity had a robust association with subsequent dementia, especially when multimorbidity occurred in middle age (6). There is, however, limited research evidence from LMICs, including China, which suffer from the greater medical and economic burden of dementia and multimorbidity. Accordingly, based on the China Health and Retirement Longitudinal Study (CHARLS), we would investigate whether multimorbidity has an association with MDs and preliminary explore the contribution of each NCD to MDs. In addition, we would also explore whether this association differed between different subgroups.

## Methods

### Study population

The study data were derived from CHARLS, an ongoing and nationally representative study conducted every 2 years focusing on community-dwelling adults aged ≥ 45 years in China. A detailed description of CHARLS has been previously reported elsewhere (26). In brief, CHARLS used a multi-stage stratified probability proportional sampling and uniformly trained investigators to collect high-quality data, including sociodemographic, lifestyle, and health-related information.

**Figure 1:**
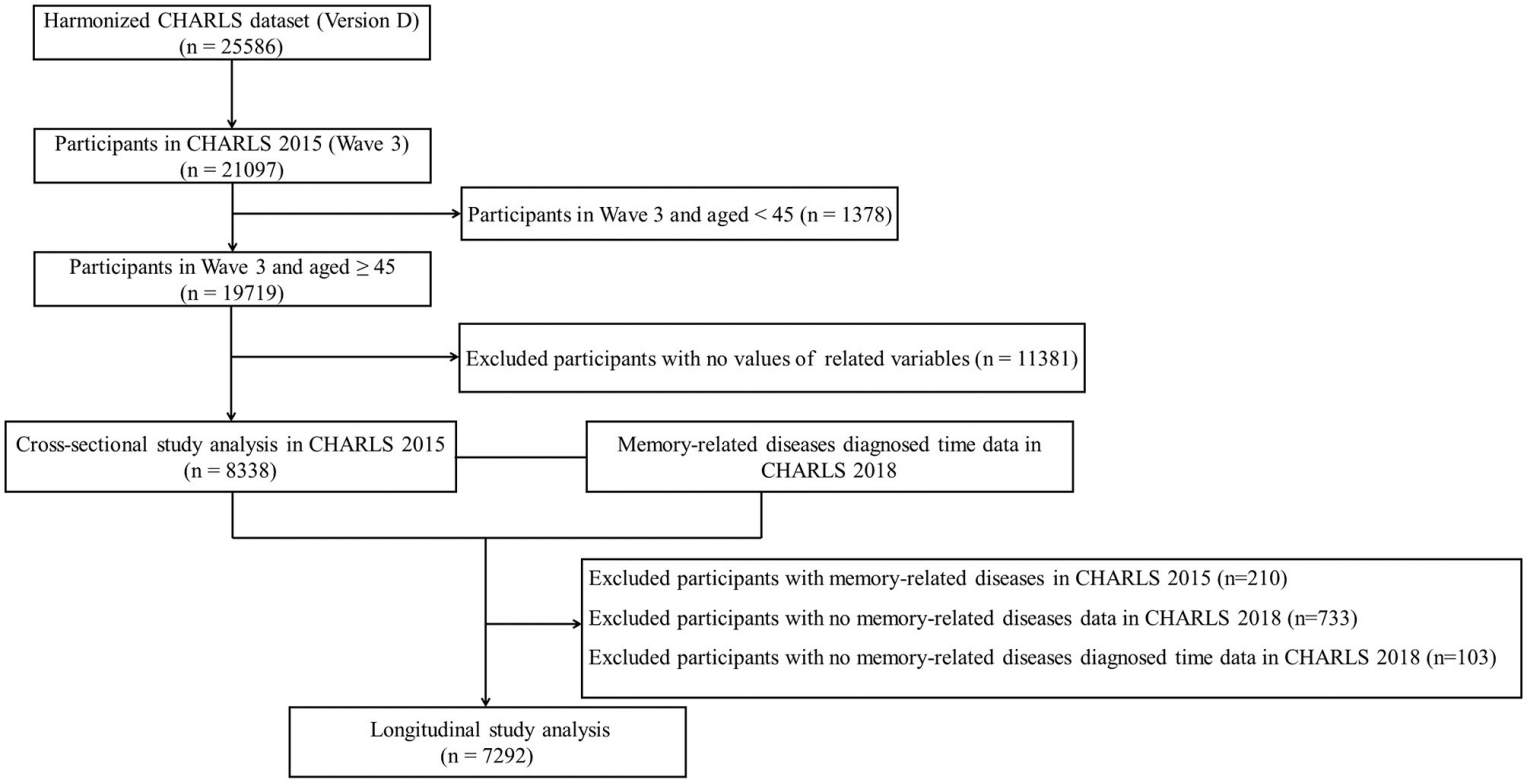
Flowchart of participants included in the study.

This study used the Harmonized CHARLS data, which were more accessible to researchers and facilitated international comparisons (26). The inclusion criteria were as follows: (1) participants in CHARLS 2015 and aged ≥ 45 years old; (2) those with information of 11 physical NCDs; and (3) those with information of the MDs status. Exclusion criteria were as follows: (1) participants aged < 45 years in CHARLS 2015; (2) missing data on 11 physical NCDs and MDs status; (3) lack of information on gender, marital status, education level, residence, socioeconomic status, body mass index (BMI), smoking status, and drinking status; and (4) missing data on MDs diagnosed time. There were two analysis datasets included in this study. The first was a cross-sectional analysis with data from CHARLS 2015, in which a total of 21,097 participants were interviewed. The second dataset of the longitudinal study further excluded participants with MDs in CHARLS 2015 (*n* = 210) and no MDs status (*n* = 733) and diagnosed time (*n* = 103) in CHARLS 2018. A detailed inclusion flowchart is shown in Figure 1.

The CHARLS was approved by the Biomedical Ethics Review Committee of Peking University, and informed consent was obtained from all participants (approval number: IRB00001052–11,015).

### NCDs and multimorbidity assessment

Similar to a previous study (16), we included 11 physical NCDs to measure multimorbidity, namely, hypertension, dyslipidemia, diabetes, heart disease, stroke, cancer, lung disease, digestive disease, liver disease, kidney disease, and arthritis. During the CHARLS, the status of NCDs was determined by self-report, which was collected by centrally trained staff using face-to-face interviews and structured questionnaires following a standard protocol. Multimorbidity was defined as the presence of two or more NCDs, and the number of NCDs indicated the severity of multimorbidity.

### MDs and diagnosed time assessment

The outcome was MDs, which included Alzheimer's disease, atrophy, and Parkinson's disease. Those participants who reported yes were defined as having MDs, and the interviewer would then ask for the first diagnosis or known time. The MDs status was assessed by the question “Have you ever been diagnosed with memory-related diseases (like Alzheimer's disease, atrophy, and Parkinson's disease) by a doctor?”. Participants who reported yes were considered to have MDs. The question would then be asked if the participants answered yes, “When was the condition (Alzheimer's disease, atrophy, or Parkinson's disease) first diagnosed or known by yourself?” The answer was taken as the time of diagnosis of MD, and then to calculate the follow-up time.

### Covariates

In CHARLS 2015, we added sociodemographic variables that included age, gender, marital status (married and others), education level, residence (urban and rural), and socioeconomic status. Health-related variables included BMI, smoking status, and drinking status. Based on the educational level characteristics of the Chinese older adults, we classified them as illiterate (no formal education illiterate), semi-literate (did not finish primary school but can read and Sishu), elementary school, middle school, high school and above (26). For socioeconomic status, we used annual per capita household consumption expenditure as a proxy (16, 27), and we divided participants into four groups based on the quartiles of annual per capita household consumption expenditure.

### Statistical analysis

Continuous variables were presented as means ± standard deviation and percentage for categorical variables. Baseline characteristics were shown as the total population and the status of MDs. The *t*-test and the chi-squared test were used to compare the group difference. In the regression analysis, we not only referred to multimorbidity as a continuous variable but also as a categorical variable and divided it into four groups: non-multimorbidity (NM, participants with no or one NCDs), multimorbidity 1 (M1, participants with two NCDs), multimorbidity 2 (M2, participants with three NCDs), and multimorbidity 3 (M3, participants with four or more NCDs). In the cross-sectional analysis, we used logistic regression analysis to estimate the association between multimorbidity and MDs. In the longitudinal study, we calculated the follow-up time based on the date of the CHARLS 2015 interview, the time of initial diagnosis of MDs, or the date of the CHARLS 2018 interview. Then, we used the Cox proportional hazards model to calculate hazard ratios (HRs) with 95% confidence intervals (CIs) to estimate the relationship between baseline multimorbidity status and incident risk of MDs. Three models were applied: Model 1, crude model; Model 2, adjusted for age, gender, marital status, education level, residence, and socioeconomic status; Model 3 additionally adjusted for BMI, smoking status, and drinking status. Stratified analysis was conducted on age, gender, residence, smoking status, drinking status, and socioeconomic status. The metrics of odds ratio (OR) and hazard ratio (HR) based on the regression models indicated that the percentage increase in the likelihood of MDs as the number of multimorbidity increased, whereas the HR value represented the increased risk of developing MDs with an increasing number of multimorbidity in subjects without baseline MDs. All statistical analyses were performed with SAS 9.4 and plotted with R version 4.1.3. A *P*-value of < 0.05 was considered statistically significant.

## Results

### Characteristics of study participants

**Figure 2:**
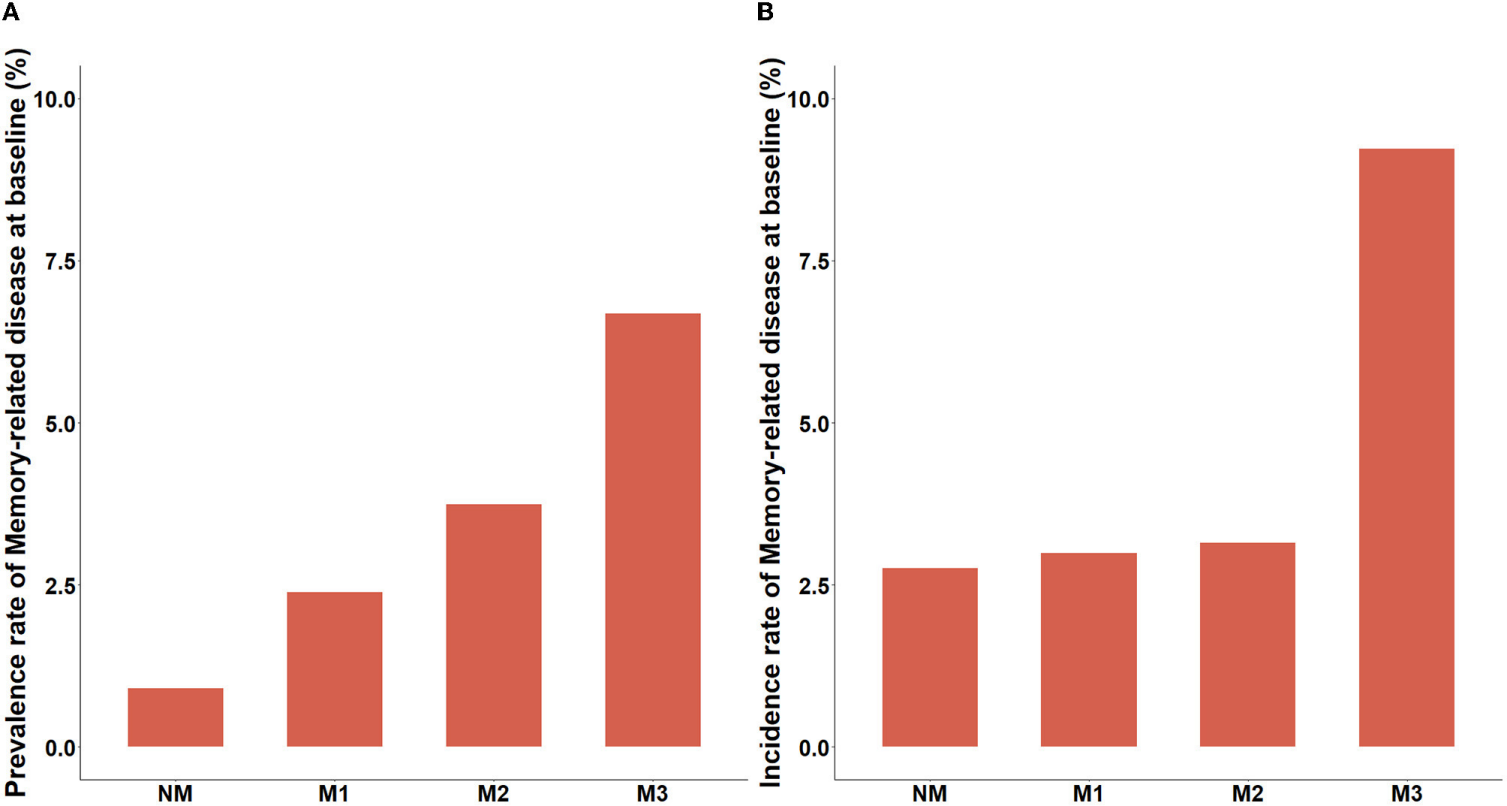
Epidemiologic feature of memory-related diseases according to the severity of multimorbidity. **(A)** Prevalence rate of memory-related diseases. **(B)** Incidence rate of memory-related diseases. NM, participants with no or one NCD; M1, participants with two NCDs; M2, participants with three NCDs; M3, participants with four or more NCDs.

Among these 8,338 middle-aged and older adults in the cross-sectional analysis, the mean age was 60.43 ± 8.94 and 48.48% were men. The average number of multimorbidity was 1.87 ± 1.60, and the prevalence of MDs was 2.52% (210/8338). Compared with the non-MDs group, those with MDs were more likely to be older (mean age, 66.62 vs. 60.27), less likely to drink (28.10 vs. 36.00%), and had a high number of NCDs (average multimorbidity number, 3.27 vs. 1.83) (all *P* < 0.05). The gender, marital status, education, residence, socioeconomic status, BMI, smoking status, and drinking status were not significantly different (Table 1). In longitudinal analysis, we presented the baseline characteristics of 7,292 participants without MDs in CHARLS 2015 (Supplementary Table S1). In addition to drink, similar results were found across MDs groups, and participants with MDs were older (mean age, 64.89 vs. 59.97) and had a higher number of NCDs (average multimorbidity number, 2.54 vs. 1.78) than participants without the incident of MDs. In addition, we compared the prevalence and incidence of MDs between the severity of multimorbidity groups, respectively. In the cross-sectional analysis, the prevalence of MDs increased from the NM group to the M3 group. Similarly, the incidence rate of MDs gradually increased with the severity of multimorbidity in longitudinal analysis (Figure 2).

**Table 1:** Baseline characteristics of participants in the cross-sectional analysis.

**Variables**	**Total**	**Memory-related diseases**	**No memory-related diseases**	**P-value**
*N*	8,338	210	8,128	
Multimorbidity count	1.87 ± 1.60	3.27 ± 1.92	1.83 ± 1.57	*P* < 0.001^*^
Age, M(SD), years	60.43 ± 8.94	66.62 ± 8.72	60.27 ± 8.89	*P* < 0.001^*^
Sex (male, %)	4,042 (48.48%)	103 (49.05%)	3,939 (48.46%)	0.87
**Marital status, %**				0.36
Married and partnered	7,466 (89.54%)	184 (87.62%)	7,282 (89.59%)	
Others	872 (10.46%)	26 (12.38%)	846 (10.41%)	
Education, %				0.43
Illiterate	1,982 (23.77%)	52 (24.76%)	1,930 (23.75%)	
Semi-illiterate	1,524 (18.28%)	44 (20.95%)	1,480 (18.21%)	
Elementary school	1,888 (22.64%)	39 (18.57%)	1,849 (22.75%)	
Middle school	1,898 (22.76%)	53 (2.79%)	1,845 (22.70%)	
High school and above	1,046 (12.54%)	22 (10.48%)	1,024 (12.60%)	
Residence (urban, %)	3,079 (36.93%)	89 (42.38%)	2,990 (36.79%)	0.10
BMI, M(SD), kg/m^2^	24.72 ± 18.98	24.74 ± 14.64	24.72 ± 19.08	0.99
Smoking, %	3,729 (44.72%)	100 (52.38%)	3,629 (44.65%)	0.39
Drinking, %	2,985 (35.80%)	59 (28.10%)	2,926 (36.00%)	0.02^*^
**Socioeconomic status**				0.12
Quartile 1 (lowest)	2,084 (24.99%)	53 (25.24%)	2,031 (24.99%)	
Quartile 2	2,081 (24.96%)	39 (18.57%)	2,042 (25.12%)	
Quartile 3	2,087 (25.03%)	55 (26.19%)	2,032 (25.00%)	
Quartile 4 (highest)	2,086 (25.02%)	63 (30.00%)	2,023 (24.89%)	

### Association of multimorbidity with MDs in the cross-sectional analysis

Overall, we found that multimorbidity and its severity were significantly associated with MDs (Table 2). Specifically, when multimorbidity was a continuous variable, we observed that the risk of MDs increased by 54% for each unit increase in the number of multimorbidities. After adjusting for sociodemographic and health-related factors, multimorbidity was also significantly associated with MDs [Model 2: OR, 1.50 (1.39–1.61); Model 3: OR, 1.49 (1.39–1.60)]. Similar results were also found when termed multimorbidity as a category variable. After adjusting for the confounding factors, participants in the M1 and M2 groups were more likely to have MDs than those in the NM group [M1 group: OR, 2.42 (1.55–3.76); M2 group: OR, 3.52 (2.23–5.56)]. It was noteworthy that, compared to the NM group, the M3 group had a 6.49-fold odds ratio of MDs [M3 group: OR, 6.49 (4.35–9.68)].

**Table 2:** Association between multimorbidity and MDs in the cross-sectional analysis.

	**Odds ratio (OR, 95%CI)**		
	**Model 1**	**Model 2**	**Model 3**
Multimorbidity count	1.54 (1.44–1.65)	1.50 (1.39–1.61)	1.49 (1.39–1.60)
NM	ref	ref	ref
M1	2.70 (1.74–4.20)	2.44 (1.57–3.80)	2.42 (1.55–3.76)
M2	4.28 (2.73–6.72)	3.56 (2.26–5.62)	3.52 (2.23–5.56)
M3	7.89 (5.32–11.70)	6.57 (4.41–9.81)	6.49 (4.35–9.68)

MDs, memory-related diseases; NM, participants with no or one NCD, M1, participants with two NCDs; M2, participants with three NCDs; M3, participants with four or more NCDs. Model 1 not adjusted; Model 2 adjusted for age, sex, marital status, education, residence, and socioeconomic status; Model 3 additionally adjusted for BMI, smoking status, and drinking status.

**Figure 3:**
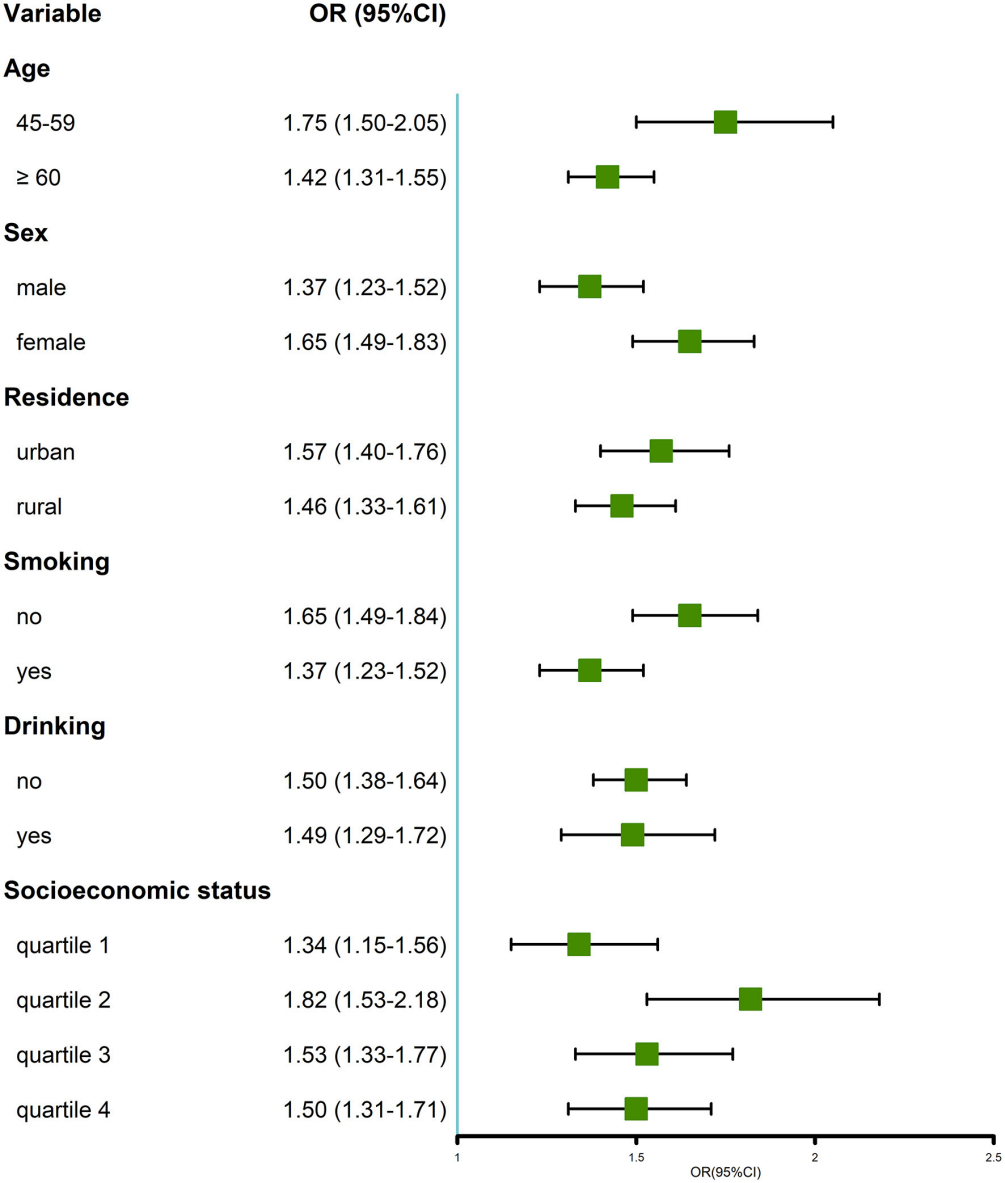
Stratified analysis of the ORs between multimorbidity and memory-related diseases.

In the stratified analysis, multimorbidity was positively associated with MDs in all subgroups (Figure 3). Particularly, when compared with their counterparts, we found that the positive association was more prominent in the subgroup of individuals aged 45–59 years, women, non-smokers, and those with quartile 2 of socioeconomic status.

### Longitudinal association between multimorbidity at baseline and incident MDs

After following up for 2.7 years, 82 cases of MDs (1.12%) were reported. In the longitudinal analysis, the incidence rate of MDs was 3.77 per 1000 person-years among all participants. The incidence rates among the multimorbidity groups were 2.76, 2.99, 3.15, and 9.22 per 1,000 person-years for the NM, M1, M2, and M3 groups, respectively. In Model 1, we found that subjects with multimorbidity were more likely to have an incident of MDs, regardless of whether the multimorbidity was as continuous [HR: 1.30 (1.15–1.46)] or categorical variable [M3 group: HR:3.34 (1.99–5.59)]. Similar results were also found in Models 2 and 3, indicating that participants with multimorbidity were more likely to have new-onset MDs than the NM group [Model 2: HR, 2.90 (1.72–4.87); Model 3: HR, 2.93 (1.74–4.96)] (Table 3).

**Table 3:** Association between multimorbidity and MDs in the longitudinal analysis.

	**Incidence Rate, per 1,000 Person-years**	**Hazard ration (HR, 95%CI)**		
		**Model 1**	**Model 2**	**Model 3**
Multimorbidity count	3.77	1.30 (1.15–1.46)	1.26 (1.12–1.42)	1.26 (1.12–1.43)
NM	2.76	ref	ref	ref
M1	2.99	1.08 (0.58–2.01)	0.99 (0.53–1.84)	0.99 (0.53–1.85)
M2	3.15	1.14 (0.54–2.40)	1.01 (0.48–2.14)	1.03 (0.49–2.18)
M3	9.22	3.34 (1.99–5.59)	2.90 (1.72–4.87)	2.93 (1.74–4.96)

MDs, memory-related diseases; NM, participants with no or one NCD, M1, participants with two NCDs; M2, participants with three NCDs; M3, participants with four or more NCDs. Model 1 no adjusted; Model 2 adjusted for age, sex, marital status, education, residence, and socioeconomic status; Model 3 additionally adjusted for BMI, smoking status, and drinking status.

**Figure 4:**
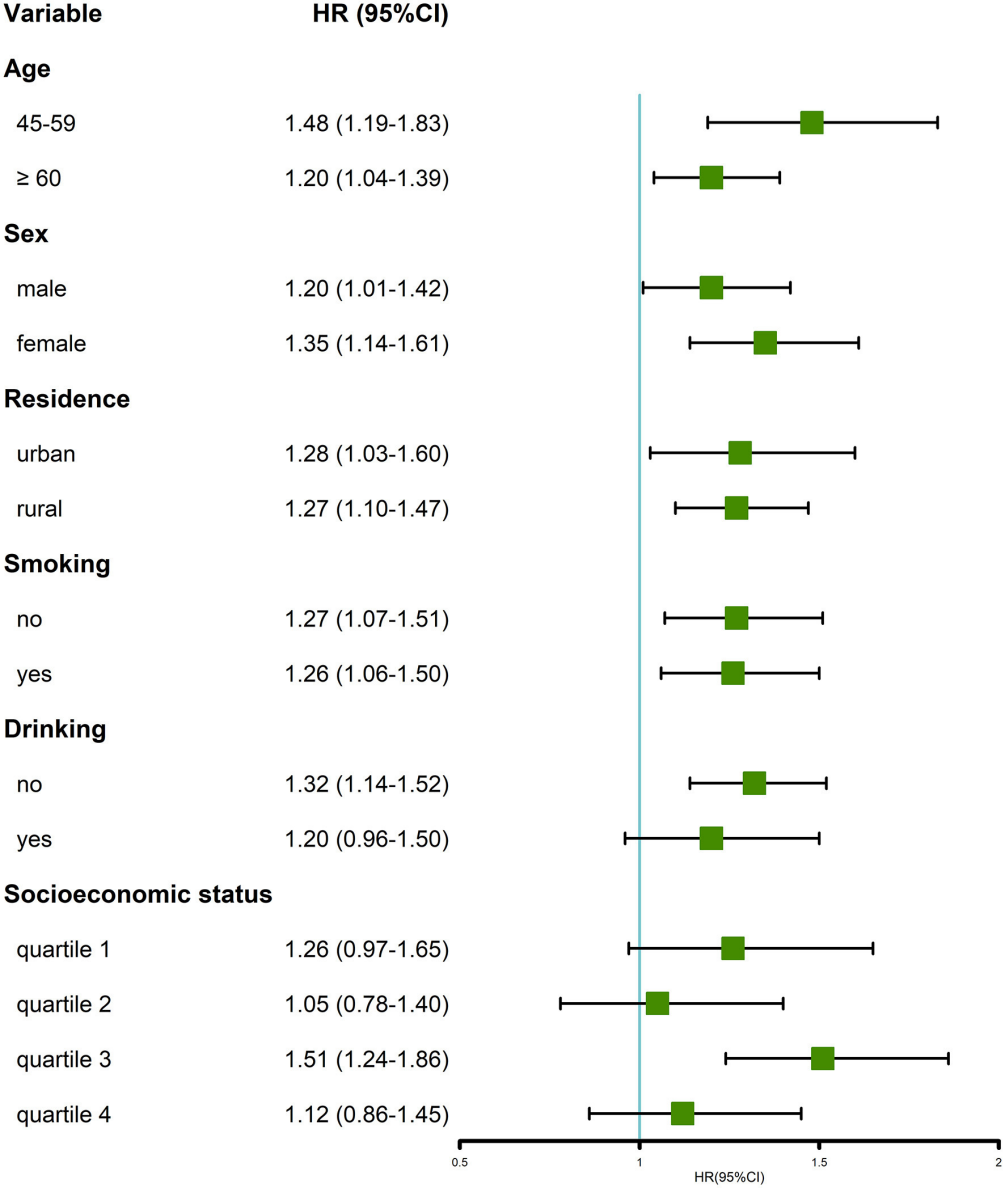
Stratified analysis of the HRs between multimorbidity and memory-related diseases.

In the stratified analysis of most of the subgroups, participants with multimorbidity were more likely to have an incidence of MDs (Figure 4). Similar to the cross-sectional analysis, the positive association was more prominent in the group of individuals aged 45–59 years and women, compared with the older (age ≥ 60 years old) and men groups, respectively. The longitudinal association was not significant in the subgroup of drink, quartiles 1, 2, and 4 of socioeconomic status.

### Cross-sectional and longitudinal associations of each NCD with MDs

To explore the extent to which each of the 11 NCDs that constituted the multimorbidity contributed to the risk of MDs, we analyzed the cross-sectional and longitudinal association between each of the 11 NCDs and MDs separately. As for the cross-sectional association, we found that, except for cancer, the other 10 NCDs all had a significantly positive association with MDs (Supplementary Table S2). In terms of effect values, the top five NCDs with the greatest risk effect on MDs were stroke [OR: 5.52 (3.73–8.17)], heart problem [OR: 2.77 (2.07–3.71)], dyslipidemia [OR: 2.60 (1.94–3.49)], kidney disease [OR: 2.28 (1.60–3.26)], and liver disease [OR: 2.06 (1.33–3.18)], whereas, only hypertension, dyslipidemia, diabetes, and kidney diseases were significantly positively associated with MDs in the longitudinal analysis, which might connect with the short follow-up time and a small number of MDs events. From the perspective of effect values, the order of these top four NCDs was diabetes [HR: 2.52 (1.46–4.33)], dyslipidemia [HR: 2.16 (1.33–3.51)], hypertension [HR: 1.99 (1.27–3.12)], and kidney disease [HR: 1.86 (1.02–3.37)] (Supplementary Table S3).

## Discussion

Among Chinese middle-aged and older adults, multimorbidity was associated with a higher risk of MDs. As for the cross-sectional association, we found that 49% increased the risk of MDs for each additional NCD and this relationship gradually strengthened with the severity of multimorbidity. Furthermore, we found that the risk of incident MDs was 26% higher for each additional NCD at baseline. Compared with the NM group, the M3 group had a nearly 3-fold higher risk of MDs. Cross-sectional and longitudinal associations remained stable across different subgroups, and this association was more prominent in middle-aged adults. To our best knowledge, this is the first study to explore the association between multimorbidity and MDs in Chinese middle-aged and older adults and found that this association was more notable in middle-aged adults.

Although many studies have focused on the association between multimorbidity and dementia or AD, this study was different and filled some gaps. Previously, cross-sectional studies have found that people with AD or dementia often had a higher proportion of multimorbidity. Regarding AD, a cross-sectional study found that multimorbidity was significantly associated with lower hippocampal volumes and lower fluorodeoxyglucose positron emission tomography standardized uptake values, which were preclinical markers of AD (28). Another related study found that patients with AD had a higher percentage of multimorbidity than controls, and higher multimorbidity was associated with greater impairment in cognition (29, 30). These studies were generally consistent with our results, although the types of MDs of interest and the study populations were different. Moreover, previous studies have focused on older adults and individual MDs. This study examined not only the older adults but also the population aged 45–59 years and found that multimorbidity was positively associated with comprehensive MDs.

In terms of the longitudinal association, a study found that neuropsychiatric, cardiovascular, and sensory impairment/cancer multimorbidity patterns were associated with dementia after followed up for approximately 8.4 years (25). Another study found that older adults with multimorbidity had a higher risk of mild cognitive impairment and dementia than people without multimorbidity (31). A recent study, which included participants aged 35 to 55 at baseline, found that multimorbidity had a robust association with subsequent dementia, and this association was more prominent in multimorbidity onset in midlife (6). These longitudinal studies further supported the relationship between multimorbidity and MDs. The above-mentioned previous studies have shown that multimorbidity was associated with MDs, but these studies were mainly implemented in high-income countries and paid less attention to multimorbidity severity and middle-aged adults. Our study, which included Chinese middle-aged and older adults, examined the cross-sectional and longitudinal associations between multimorbidity and MDs.

For these individual NCDs that constituted the multimorbidity, we found that stroke, cancer, heart problem, dyslipidemia, and kidney diseases had a stronger cross-sectional relationship with MDs. There were some relevant studies in favor of this relationship, and there would be some mechanisms involved (32). A study reported that stroke was associated with cognitive impairment and dementia in older adults, and the six RNA-binding protein (RBP) genes (POLR2F, DYNC1H1, SMAD9, TRIM21, BRCA1, and ERI1) might participate in the process by mediating the hypoxic responses and angiogenesis (33). Regarding cardiovascular diseases and memory function, studies jointly found that better cardiovascular health is associated with lower memory decline and risk of cognitive impairment (34, 35). Another cohort study found that decreased numbers of optimal cardiovascular health metrics were associated with a higher risk of dementia (35). In a meta-analysis study, which included more than 50 000 participants, kidney disease was associated with cognitive impairment (36). In the longitudinal association between individual NCDs and MDs, only diabetes, dyslipidemia, hypertension, and kidney disease remained statistically significant. This might be because the follow-up time was short and there were only a few MDs events. We unified the analysis of 11 NCDs because this situation was closer to the real-world problem of middle-aged and older adults (6). Even though individual NCDs could affect the risk of memory function, studies of multimorbidity suggested that the aggregation of NCDs had a specific cumulative effect that might accelerate cognitive decline and increase dementia risk (31, 37). This was consistent with our study, which showed that participants with four or more NCDs had more significant effect values with MDs. This process might be involved in the inflammatory process (25) and the interaction between and/or cumulative with medications prescribed (31).

Our study has several strengths. First, this study examined the relationship between multimorbidity, rather than individual NCDs, and MDs among Chinese middle-aged and older adults, which was more consistent with the real-world situation. Because the subjects in this study were from a nationally representative study, our findings could be generalized to general adults. Second, the present study further analyzed the longitudinal association and the cross-sectional association. In addition, our findings tentatively suggested that the severity of multimorbidity had a cumulative effect on MDs. There are also some limitations to this study. First, the diagnosis of NCDs and MDs was self-reported rather than based on medical records, which might produce some misjudgment. A study reported that there was a 75% accuracy in self-reported disease (38). Second, since we lacked data on different MDs types, we were unable to provide a more explicit analysis of the MDs types. Third, this study was a retrospective analysis, so we cannot make causal inferences based on it, and the follow-up time was short. Future long-time cohort studies are needed to pinpoint this causal effect. Despite these limitations, our study found a risk relationship between multimorbidity, a natural condition in middle-aged and older adults, and MDs. This indicated that physicians and the public should pay more attention to multimorbidity patients, especially those who commonly have four or more NCDs, and to prevent the risk of future MDs in advance.

## Conclusion

Multimorbidity is becoming more common, and the age of onset is getting younger. Simultaneously, given the lack of effective treatments for memory decline and its impact on individuals and society, it is imperative to identify the primary risk factors to prevent it in advance. Our study revealed that multimorbidity was associated with MDs among Chinese middle-aged and older adults, and this association was more prominent in middle-aged adults. This relationship gradually strengthened with the severity of multimorbidity. This finding may help reduce the risk of MDs and the medical and financial burden by implementing early prevention strategies for people with a high risk of MDs.

## Data availability statement

The original contributions presented in the study are included in the article/Supplementary material, further inquiries can be directed to the corresponding author.

## Author contributions

All authors listed have made a substantial, direct, and intellectual contribution to the work and approved it for publication.

## References

[1] Grande G Qiu C Fratiglioni L. Prevention of dementia in an ageing world: evidence and biological rationale. Ageing Res Rev. (2020) 64:101045. 10.1016/j.arr.2020.101045 32171784

[2] WHO. World Report on Ageing and Health. (2020) Available online at: https://scholar.google.com/scholar_lookup?title=World%20Report%20on%20Ageing%20and%20Health&author=WHO&publication_year=2015

[3] Collaborators GD. Global, regional, and national burden of Alzheimer's disease and other dementias, 1990-2016: a systematic analysis for the global burden of disease study 2016. Lancet Neurol. (2019) 18:88–106. 10.1016/S1474-4422(18)30403-4 30497964 PMC6291454

[4] World Alzheimer Report 2022. (2020) Available online at: https://www.alzint.org/u/World-Alzheimer-Report-2022.pdf

[5] Ren R Qi J Lin S Liu X Yin P Wang Z. The China Alzheimer report 2022. Gen Psychiatr. (2022) 35:e100751. 10.1136/gpsych-2022-100751 35372787 PMC8919463

[6] Ben Hassen C Fayosse A Landré B Raggi M Bloomberg M Sabia S. Association between age at onset of multimorbidity and incidence of dementia: 30 year follow-up in Whitehall II prospective cohort study. BMJ. (2022) 376:e068005. 10.1136/bmj-2021-068005 35110302 PMC9086721

[7] Kalia LV Lang AE. Parkinson's disease. Lancet. (2015) 386:896–912. 10.1016/S0140-6736(14)61393-3 25904081

[8] Yu B Steptoe A Chen Y Jia X. Social isolation, rather than loneliness, is associated with cognitive decline in older adults: the china health and retirement longitudinal study. Psychol Med. (2021) 51:2414–21. 10.1017/S0033291720001014 32338228

[9] Vetrano DL Rizzuto D Calderón-Larrañaga A Onder G Welmer AK Bernabei R. Trajectories of functional decline in older adults with neuropsychiatric and cardiovascular multimorbidity: a Swedish cohort study. PLoS Med. (2018) 15:e1002503. 10.1371/journal.pmed.1002503 29509768 PMC5839531

[10] Jia J Wei C Chen S Li F Tang Y Qin W. The cost of Alzheimer's disease in China and re-estimation of costs worldwide. Alzheimers Dement. (2018) 14:483–91. 10.1016/j.jalz.2017.12.006 29433981

[11] Global regional and and national age-sex-specific mortality for 282 causes of death in 195 countries and territories 1980-2017: 1980-2017: a systematic analysis for the global burden of disease study 2017. Lancet. (2018) 392:1736–88. 10.1016/S0140-6736(18)32203-7 30496103 PMC6227606

[12] Tinetti ME Fried TR Boyd CM. Designing health care for the most common chronic condition–multimorbidity. JAMA. (2012) 307:2493–4. 10.1001/jama.2012.5265 22797447 PMC4083627

[13] Barnett K Mercer SW Norbury M Watt G Wyke S Guthrie B. Epidemiology of multimorbidity and implications for health care, research, and medical education: a cross-sectional study. Lancet. (2012) 380:37–43. 10.1016/S0140-6736(12)60240-2 22579043

[14] Kingston A Robinson L Booth H Knapp M Jagger C. Projections of multi-morbidity in the older population in England to 2035: estimates from the population ageing and care simulation (PACSim) model. Age Ageing. (2018) 47:374–80. 10.1093/ageing/afx201 29370339 PMC5920286

[15] Sum G Hone T Atun R Millett C Suhrcke M Mahal A. Multimorbidity and out-of-pocket expenditure on medicines: a systematic review. BMJ Glob Health. (2018) 3:e000505. 10.1136/bmjgh-2017-000505 29564155 PMC5859814

[16] Zhao Y Atun R Oldenburg B McPake B Tang S Mercer SW. Physical multimorbidity, health service use, and catastrophic health expenditure by socioeconomic groups in China: an analysis of population-based panel data. Lancet Glob Health. (2020) 8:e840–9. 10.1016/S2214-109X(20)30127-3 32446349 PMC7241981

[17] Stubbs B Koyanagi A Veronese N Vancampfort D Solmi M Gaughran F. Physical multimorbidity and psychosis: comprehensive cross sectional analysis including 242,952 people across 48 low- and middle-income countries. BMC Med. (2016) 14:189. 10.1186/s12916-016-0734-z 27871281 PMC5118890

[18] Arokiasamy P Uttamacharya U Jain K Biritwum RB Yawson AE Wu F. The impact of multimorbidity on adult physical and mental health in low- and middle-income countries: what does the study on global ageing and adult health (SAGE) reveal? BMC Med. (2015) 13:178. 10.1186/s12916-015-0402-8 26239481 PMC4524360

[19] Beard JR Officer A de Carvalho IA Sadana R Pot AM Michel JP. The world report on ageing and health: a policy framework for healthy ageing. Lancet. (2016) 387:2145–54. 10.1016/S0140-6736(15)00516-4 26520231 PMC4848186

[20] Shang Y Fratiglioni L Marseglia A Plym A Welmer AK Wang HX. Association of diabetes with stroke and post-stroke dementia: A population-based cohort study. Alzheimers Dement. (2020) 16:1003–12. 10.1002/alz.12101 32489021

[21] Abell JG Kivimäki M Dugravot A Tabak AG Fayosse A Shipley M. Association between systolic blood pressure and dementia in the Whitehall II cohort study: role of age, duration, and threshold used to define hypertension. Eur Heart J. (2018) 39:3119–25. 10.1093/eurheartj/ehy288 29901708 PMC6122131

[22] Ding M Fratiglioni L Johnell K Santoni G Fastbom J Ljungman P. Atrial fibrillation, antithrombotic treatment, and cognitive aging: a population-based study. Neurology. (2018) 91:e1732–e40. 10.1212/WNL.0000000000006456 30305443 PMC6251601

[23] Calderón-Larrañaga A Vetrano DL Ferrucci L Mercer SW Marengoni A Onder G. Multimorbidity and functional impairment-bidirectional interplay, synergistic effects and common pathways. J Intern Med. (2019) 285:255–71. 10.1111/joim.12843 30357990 PMC6446236

[24] Bunn F Burn AM Goodman C Rait G Norton S Robinson L. Comorbidity and dementia: a scoping review of the literature. BMC Med. (2014) 12:192. 10.1186/s12916-014-0192-4 25358236 PMC4229610

[25] Grande G Marengoni A Vetrano DL Roso-Llorach A Rizzuto D Zucchelli A. Multimorbidity burden and dementia risk in older adults: The role of inflammation and genetics. Alzheimers Dement. (2021) 17:768–76. 10.1002/alz.12237 33403740 PMC8247430

[26] *Harmonized CHARLS Documentation VERSION D (2011-2018)*. (2021) Available online at: https://charls.charlsdata.com/Public/ashelf/public/uploads/document/harmonized_charls/application/Harmonized_CHARLS_D.pdf

[27] Zhao Y Atun R Anindya K McPake B Marthias T Pan T. Medical costs and out-of-pocket expenditures associated with multimorbidity in China: quantile regression analysis. BMJ Glob Health. (2021) 6:2. 10.1136/bmjgh-2020-004042 33632770 PMC7908909

[28] Mendes A Tezenas du Montcel S Levy M Bertrand A Habert MO Bertin H. Multimorbidity is associated with preclinical Alzheimer's disease neuroimaging biomarkers dement. Geriatr Cogn Disord. (2018) 45:272–81. 10.1159/000489007 29953971

[29] Wang JH Wu YJ Tee BL Lo RY. Medical comorbidity in alzheimer's disease: a nested case-control study. J Alzheimers Dis. (2018) 63:773–81. 10.3233/JAD-170786 29660933

[30] Doraiswamy PM Leon J Cummings JL Marin D Neumann PJ. Prevalence and impact of medical comorbidity in Alzheimer's disease. J Gerontol A Biol Sci Med Sci. (2002) 57:M173–7. 10.1093/gerona/57.3.M173 11867654

[31] Vassilaki M Aakre JA Cha RH Kremers WK St Sauver JL Mielke MM. Multimorbidity and risk of mild cognitive impairment. J Am Geriatr Soc. (2015) 63:1783–90. 10.1111/jgs.13612 26311270 PMC4607039

[32] Winblad B Amouyel P Andrieu S Ballard C Brayne C Brodaty H. Defeating Alzheimer's disease and other dementias: a priority for european science and society. Lancet Neurol. (2016) 15:455–532. 10.1016/S1474-4422(16)00062-4 26987701

[33] Lin W Wang Q Chen Y Wang N Ni Q Qi C. Identification of a 6-RBP gene signature for a comprehensive analysis of glioma and ischemic stroke: cognitive impairment and aging-related hypoxic stress. Front Aging Neurosci. (2022) 14:951197. 10.3389/fnagi.2022.951197 36118697 PMC9476601

[34] Gardener H Wright CB Dong C Cheung K DeRosa J Nannery M. Ideal cardiovascular health and cognitive aging in the northern manhattan study. J Am Heart Assoc. (2016) 5:e002731. 10.1161/JAHA.115.002731 26984255 PMC4943249

[35] Samieri C Perier MC Gaye B Proust-Lima C Helmer C Dartigues JF. Association of cardiovascular health level in older age with cognitive decline and incident dementia. JAMA. (2018) 320:657–64. 10.1001/jama.2018.11499 30140876 PMC6142948

[36] Berger I Wu S Masson P Kelly PJ Duthie FA Whiteley W. Cognition in chronic kidney disease: a systematic review and meta-analysis. BMC Med. (2016) 14:206. 10.1186/s12916-016-0745-9 27964726 PMC5155375

[37] Song X Mitnitski A Rockwood K. Nontraditional risk factors combine to predict Alzheimer's disease and dementia. Neurology. (2011) 77:227–34. 10.1212/WNL.0b013e318225c6bc 21753161 PMC3136058

[38] Xie W Zheng F Yan L Zhong B. Cognitive decline before and after incident coronary events. J Am Coll Cardiol. (2019) 73:3041–50. 10.1016/j.jacc.2019.04.019 31221251

