# Supplementary material for: Association between multimorbidity and memory-related diseases among middle-aged and older adults: Evidence from the China Health and Retirement Longitudinal Study
Source: Front Public Health. 2023 Mar 17;11:1115207. doi: 10.3389/fpubh.2023.1115207 (PMC10065148; doi:10.3389/fpubh.2023.1115207)
Supplement: Supplementary file 1 [file Data_Sheet_1.pdf]

Supplemental File

Journal: Frontiers in Public Health

Title: Association between multimorbidity and memory-related diseases among middle-aged and older adult: Evidence from the China Health and Retirement Longitudinal Study

Table S1 Baseline characteristics of participants in the longitudinal analysis

Variables	Total	Memory-related diseases	No memory-related diseases	*P* value
N	7292	82	7210	
Multimorbidity count	1.79±1.54	2.54±1.94	1.78±1.54	0.0008*
Age, M(SD), years	60.03±8.72	64.89±8.20	59.97±8.71	*P* <0.0001*
Sex (male, %)	3468 (47.56%)	45 (54.88%)	3423 (47.48%)	0.182
Marital status, %				0.487
married and partnered	6569 (90.09%)	72 (87.80%)	6497 (90.11%)	
others	723 (9.91%)	10 (12.20%)	713 (9.89%)	
Education, %				0.374
illiterate	1758 (24.11%)	25 (30.49%)	1733 (24.04%)	
semi-illiterate	1310 (17.96%)	14 (17.07%)	1296 (17.98%)	
elementary school	1649 (22.61%)	21 (25.61%)	1628 (22.58%)	
middle school	1670 (22.90%)	12 (14.63%)	1658 (23.00%)	
high school and above	905 (12.41%)	10 (12.20%)	895 (12.41%)	
Residence (urban, %)	2614 (35.85%)	25 (30.49%)	2589 (35.91%)	0.309
BMI, M(SD), kg/㎡	24.83±19.95	23.86±4.21	24.84±20.06	0.061
Smoking, %	3205 (43.95%)	42 (51.22%)	3163 (43.87%)	0.182
Drinking, %	2622 (35.96%)	30 (36.59%)	2592 (35.95%)	0.905
Socioeconomic status				0.912
quartile 1 (lowest)	1839 (25.22%)	20 (24.39%)	1819 (25.23%)	
quartile 2	1838 (25.21%)	19 (23.17%)	2819 (25.23%)	
quartile 3	1804 (24.74%)	23 (28.05%)	1781 (24.70%)	
quartile 4 (highest)	1811 (24.84%)	20 (24.39%)	1791 (24.84%)	

Table S2 Logistic regression of each non-communicable disease and memory-related diseases

	The odds ratio of Memory-related diseases		
	Model 1	Model 2	Model 3
Stroke	6.97 (4.78-10.16)	5.64 (3.82-8.33)	5.52 (3.73-8.17)
Cancer	1.61 (0.65-3.99)	1.44 (0.57-3.60)	3.38 (0.55-3.46)
Heart problem	3.44 (2.59-4.55)	2.80 (2.10-3.75)	2.77 (2.07-3.71)
Dyslipidemia	2.73 (2.05-3.63)	2.60 (1.94-3.49)	2.60 (1.94-3.49)
Kidney disease	2.38 (1.68-3.37)	2.29 (1.60-3.27)	2.28 (1.60-3.26)
Liver disease	1.97 (1.29-3.02)	2.08 (1.35-3.21)	2.06 (1.33-3.18)
Hypertension	2.48 (1.89-3.27)	2.03 (1.53-2.69)	2.02 (1.53-2.68)
Arthritis	2.00 (1.52-2.64)	1.91 (1.43-2.54)	1.90 (1.43-2.53)
Digestive disease	1.75 (1.33-2.31)	1.88 (1.42-2.49)	1.87 (1.41-2.47)
Diabetes	2.14 (1.49-3.06)	1.81 (1.25-2.62)	1.79 (1.24-2.59)
Lung disease	1.75 (1.26-2.44)	1.52 (1.08-2.13)	1.48 (1.05-2.09)

Model 1 no adjusted; Model 2 adjusted for age, sex, marital status, education, residence and socio-economic status; Model 3 additionally adjusted for BMI, smoking and drinking.

Table S3 Cox regression of each non-communicable disease and memory-related diseases

	The hazard ratio of Memory-related diseases		
	Model 1	Model 2	Model 3
Diabetes	2.59 (1.52-4.42)	2.50 (1.45-4.29)	2.52 (1.46-4.33)
Dyslipidemia	2.05 (1.28-3.28)	2.13 (1.32-3.44)	2.16 (1.33-3.51)
Hypertension	2.21 (1.43-3.41)	1.93 (1.25-2.99)	1.99 (1.27-3.12)
Cancer	1.98 (0.49-8.07)	1.91 (0.47-7.80)	1.92 (0.47-7.82)
Kidney disease	1.98 (1.10-3.59)	1.86 (1.03-3.37)	1.86 (1.02-3.37)
Heart problem	1.70 (1.03-2.82)	1.54 (0.92-2.57)	1.54 (0.92-2.57)
Digestive disease	1.49 (0.96-2.31)	1.52 (0.97-2.36)	1.51 (0.97-2.36)
Lung disease	1.46 (0.84-2.56)	1.23 (0.70-2.16)	1.22 (0.69-2.15)
Arthritis	1.20 (0.78-1.86)	1.11 (0.71-1.72)	1.11 (0.71-1.72)
Stroke	1.34 (0.42-4.24)	1.09 (0.34-3.45)	1.09 (0.34-3.46)
Liver disease	0.19 (0.03-1.33)	0.19 (0.03-1.36)	0.19 (0.03-1.35)

Model 1 no adjusted; Model 2 adjusted for age, sex, marital status, education, residence and socio-economic status; Model 3 additionally adjusted for BMI, smoking and drinking.
